# Structural disorder in metallic glass-forming liquids

**DOI:** 10.1038/srep27708

**Published:** 2016-06-09

**Authors:** Shao-Peng Pan, Shi-Dong Feng, Li-Min Wang, Jun-Wei Qiao, Xiao-Feng Niu, Bang-Shao Dong, Wei-Min Wang, Jing-Yu Qin

**Affiliations:** 1 College of Materials Science and Engineering, Taiyuan University of Technology, Taiyuan, 030024, China; 2 Shanxi key laboratory of advanced magnesium-based materials, Taiyuan University of Technology, Taiyuan, 030024, China; 3 State Key Laboratory of Metastable Materials Science and Technology, Yanshan University, Qinhuangdao, 066004, China; 4 Advanced Technology & Materials Co., Ltd., China Iron & Steel Research Institute Group, Beijing, 100081, China; 5 Key Laboratory for Liquid-Solid Structural Evolution and Processing of Materials (Ministry of Education), Shandong University, Jinan 250061, China

## Abstract

We investigated structural disorder by a new structural parameter, quasi-nearest atom (QNA), in atomistic configurations of eight metallic glass-forming systems generated through molecular dynamics simulations at various temperatures. Structural analysis reveals that the scaled distribution of the number of QNA appears to be an universal property of metallic liquids and the spatial distribution of the number of QNA displays to be clearly heterogeneous. Furthermore, the new parameter can be directly correlated with potential energy and structural relaxation at the atomic level. Some straightforward relationships between QNA and other properties (per-atom potential energy and α-relaxation time) are introduced to reflect structure-property relationship in metallic liquids. We believe that the new structural parameter can well reflect structure disorder in metallic liquids and play an important role in understanding various properties in metallic liquids.

Full understanding the atomic structure of metallic melts is helpful on the production of metallic materials^1^,^2^,^3^. However, the identification and successive analysis of atomic structure in metallic liquids is a formidable scientific challenge which is attracting constant interest^4^,^5^,^6^. Compared with the metallic crystals, the atomic structure of metallic melts is more disordered, which are closely correlated with various properties in metallic melts^7^. Due to a lack of the long-range order in metallic melts, it is difficult to reflect all the aspect of the atomic structure in metallic melts by several structural parameters. How to better describe the atomic structure, especially to better reflect the structure-property relationship in metallic liquids and glasses becomes the research hotspot of many physical scientists.

Previous studies have studied the structure-property relationship in metallic liquids and glasses. Icosahedral clusters have been proved to play an important role in structure-property relationship in metallic liquids and glasses^8^, however, in some systems icosahedral clusters are absent^9^. The Debye-Waller factor has been quite successful for predicting the relative long-time dynamical heterogeneity^10^. The localized soft mode was used to search origin of dynamic heterogeneity in liquids^11^ and deformation in metallic glasses^12^. However, both of the two characterizations cannot provide a clear picture of local atomic structure. Many works reveals that there exists some regions, where the atomic packing is rather loose or dense, in metallic glasses and these regions are closely correlated with the properties in metallic glasses^7^. However, the present structure parameters cannot effectively identify the degree of atomic packing. The free volume concept^13^,^14^,^15^ might be one choice to describe the degree of atomic packing. However, it is impossible to measure the free volume rigorously because an atom does not have an indefinite volume^16^. Besides that, the value of free volume cannot provide any direct structure information. Therefore, although there has been some significant progress to characterize local structure, an effective parameter to directly describe and quantify the local packing in metallic liquids is still needed.

In previous work, we propose a new structural parameter, quasi-nearest atom (QNA), and found that QNA shows close correlation with dynamic heterogeneity in a metallic liquid^17^. We think that QNA can be used to describe local packing in metallic liquids. In this work, we will investigate structural disorder by QNA and its role in structure-property relationship in metallic liquids.

## Results and Discussion

### The temperature dependence of QNA

**Figure 1:**
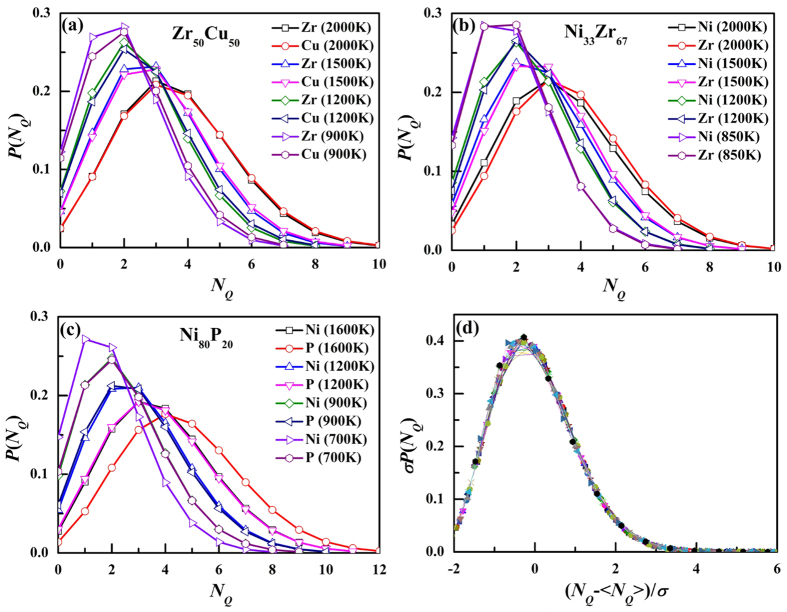
The temperature dependence of the distributions of *N*_*Q*_ in (**a**) Zr_50_Cu_50_, (**b**)Ni_33_Zr_67_ and (**c**) Ni_80_P_20_, (**d**) the distributions of *N*_*Q*_, shifted by the average value <*N*_*Q*_> and scaled by the standard deviation *σ* for those in (**a–c**).

Figure 1 shows the distribution of the number of QNAs (*N*_*Q*_) in three model systems. Figure 1(a) displays the distributions in Zr_50_Cu_50_, a well-known metallic system with icosahedral short range order (ISRO)^18^. At the temperature of 2000 K, 1500 K, 1200 K and 900 K, the distributions of *N*_*Q*_ around Zr and Cu are almost the same, indicating the similar denseness of atomic packing around Zr and Cu atoms. At 2000 K, the distributions have a peak at *N*_*Q*_~3. As temperature decreases, the positions of the peaks move to smaller *N*_*Q*_, suggesting that the atomic packing of the system becomes denser as temperature decreases. Figure 1(b) shows the distribution in Ni_33_Zr_67_, a metallic system with few ISRO^9^. Similar to Zr_50_Cu_50_, the distribution of *N*_*Q*_ around the two components are almost the same and the positions of the peaks move to smaller *N*_*Q*_ as temperature decreases. As shown in Fig. 1(c), the distributions of *N*_*Q*_ in Ni_80_P_20_, a metal-metalloid system^19^, are a little different from those in Zr_50_Cu_50_ and Ni_33_Zr_67_. At each temperature, the distributions for Ni and P are quite different. The positions of peaks for P are located at larger *N*_*Q*_. This fact indicates that the atomic packing around P atoms is much looser than that around Ni atoms, which is reasonable for metalloid element. Although the distributions of *N*_*Q*_ change as systems and temperatures, their shapes are similar. Figure 1(d) displays the distributions shifted by the average value <*N*_*Q*_> and scaled by the standard deviation *σ* for those in (a–c). This leads to the striking result that the scaled distribution of *N*_*Q*_ appears to be universal, which is similar to the Voronoi cell volume and asphericity^14^. The scaling of *P*(*N*_*Q*_) suggests that there might exist a single underlying geometrical structure of the system for metallic liquids with embedded atom method (EAM) potentials, and that system specifics, such as temperature and density, are absorbed into the average and variance of the distribution. The universal distribution of *N*_*Q*_ might be the basic property in metallic liquids. Further work should be done in more systems with other potentials for confirmation.

**Figure 2:**
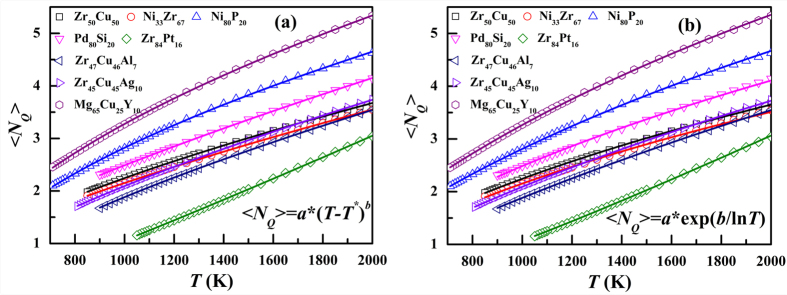
The temperature dependence of <*N*_*Q*_> in eight systems, the solid curves are the fittings with (**a**) equation (1) and (**b**) equation (2).

Figure 2 shows the temperature dependence of the average *N*_*Q*_, <*N*_*Q*_>, for eight systems. As temperature decreases, <*N*_*Q*_> decreases, suggesting that the atomic packing of the system becomes denser and denser. At high temperatures, <*N*_*Q*_> displays a linear temperature dependence. As temperature decreases, the linear correlation is deviated. Here, we proposed a power law of <*N*_*Q*_> ~ (*T* − T^*^)^*b*^ to fit <*N*_*Q*_> as a function of temperature shown in Fig. 2(a):


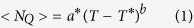


The simulated data are fitted very well by the power-law function and the statistical correlation parameter R^2^ is better than 0.99. At T^*^, <*N*_*Q*_> should be zero, which means the system has no structural “defect” and should be the ideal glass. Thus, T^*^ should be the ideal glass transition temperature. However, as shown in Table 1, T^*^is much lower than the glass transition temperature *T*_g_ and even lower than Vogel-Fulcher-Tammann (VFT) temperature^20^ for relaxation time, which is thought to be the ideal glass transition temperature. Therefore, although the fitting seems very well, equation (1) might not reflect all the nature of the temperature dependence of *N*_*Q*_. Similar to viscosity or relaxation time which can be fitted by many equations, <*N*_*Q*_> might have other good fitting equations. Figure 2(b) displays another fitting equation:


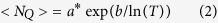


which has only two parameters, *a*, and *b*. It can be found the simulated data are also fitted very well by the equation. However, equation (2) cannot reflect the existence of ideal glass transition. More work will be done to search more reasonable fitting equations in the future.

### Spatial distribution of QNA

**Figure 3:**
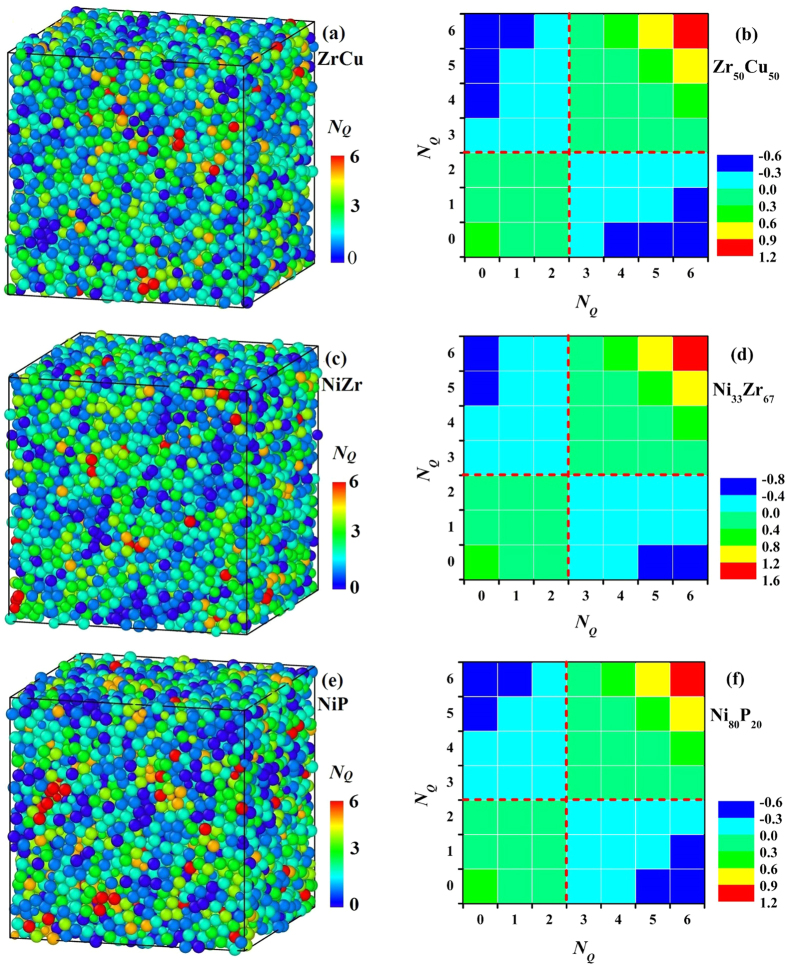
Atom configurations with atoms colored by their *N*_*Q*_ for (**a**) Zr_50_Cu_50_, (**c**) Ni_33_Zr_67_ and (**e**) Ni_80_P_20_ at *T*_g_ + 150 K. The matrix of spatial correlation index *C*_*ij*_ of atoms with different *N*_*Q*_ in (**b**) Zr_50_Cu_50_, (**d**) Ni_33_Zr_67_ and (**f**) Ni_80_P_20_ at *T*_g_ + 150 K.

Figure 3(a,c,e) display the atomic configurations with atoms colored by their *N*_*Q*_ for Zr_50_Cu_50_, Ni_33_Zr_67_ and Ni_80_P_20_ MGs at *T*_g_ + 150 K. It can be seen that the distribution of *N*_*Q*_ shows clear spatial heterogeneity. The atoms with less *N*_*Q*_ or more *N*_*Q*_ tends to be located together. To quantify the spatial arrangement of *N*_*Q*_, we calculated a nearest-neighbor correlation index^21^,^22^, 
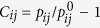
, where *p*_*ij*_ and 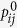 are the probability of atoms with the *N*_*Q*_ types *i* and *j* being the nearest neighbors in a structure model and a structure in which the distributions of atoms with different *N*_*Q*_ are spatially uncorrelated, respectively. Therefore, the positive and negative values indicate a preference and an avoiding of atoms with the *N*_*Q*_ types *i* and *j* being nearest neighbors, respectively. Figure 3(b,d,f) show the matrix of spatial correlation index *C*_*ij*_ of atoms in the liquid structure of Zr_50_Cu_50_, Ni_33_Zr_67_ and Ni_80_P_20_, respectively. Generally in all the three systems, all the atoms are naturally divided into two groups. One is the atoms with small *N*_*Q*_ (*N*_*Q*_ ≤ 2) and the other one is the atoms with large *N*_*Q*_ (*N*_*Q*_ > 2). When atoms belong to the same group, *C*_*ij*_ is always positive, indicating that they intend to be nearest neighbors. When atoms belong to different groups, *C*_*ij*_ is negative, suggesting that they avoid being nearest neighbors. This fact indicates that QNA does have spatial heterogeneities.

### Correlating QNA with potential energy

**Figure 4:**
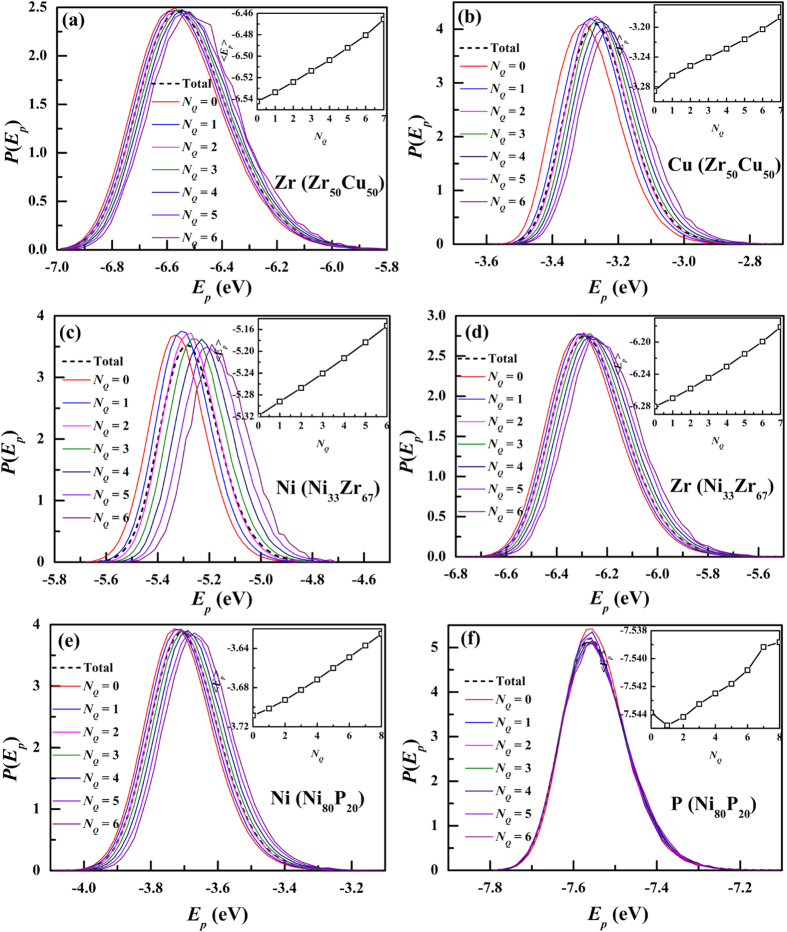
The distribution of potential energy for (**a**) Zr and (**b**) Cu in Zr_50_Cu_50_, (**c**) Ni and (**d**) Zr in Ni_33_Zr_67_ as well as (**e**) Ni and (**f**) P in Ni_80_P_20_ with different *N*_*Q*_ at Tg + 150 K. Insets are the *N*_*Q*_ dependence of the average per-atom potential energy.

In Fig. 4, we investigated the correlation between potential energy and *N*_*Q*_ in three models. The distribution of atomic potential energy with different *N*_*Q*_for Zr and Cu in Zr_50_Cu_50_ is shown in Fig. 4(a,b), respectively. It can be seen that the distributions with different *N*_*Q*_ have large overlaps, indicating the correlation between *N*_*Q*_and atomic potential energy is not a one-to-one correspondence. Since the cutoff distance of potential energy is 6.5 Å, much larger that the scale of *N*_*Q*_, it is reasonable for the large overlaps. However, as shown in the insets of Fig. 4(a,b), atoms with larger *N*_*Q*_ have less negative per-atom potential energy shown. In this respect, *N*_*Q*_ plays an key role in the correlation between local structure and potential energy. This fact suggests that atoms with larger *N*_*Q*_tend to have lower thermodynamic stability. Figure 4(c,d) shows the results in Ni_33_Zr_67_ and they are similar to those in Zr_50_Cu_50_. However, the results in Ni_80_P_20_, as shown in Fig. 4(e,f) display different features. The distribution of potential energy for Ni with different *N*_*Q*_ is similar to those in Fig. 4(a–d) while that for P is quite different. The distributions of potential energy for P with different *N*_*Q*_ have so large overlaps that all the curves seen to be coincided, suggesting that the correlation between *N*_*Q*_and atomic potential energy for P atoms is rather weak. Why P atoms show quite different feature? It can be seen in Fig. 1(c) that the atomic packing around P atoms is rather loose. Therefore, the correlation between potential energy and atomic packing might be strong in dense-packing systems such as metallic systems and be weak in the loose-packing systems. That might be the reason why the correlation between *N*_*Q*_and atomic potential energy for P atoms is rather weak. Therefore, *N*_*Q*_ is more applicable to dense-packing systems.

**Figure 5:**
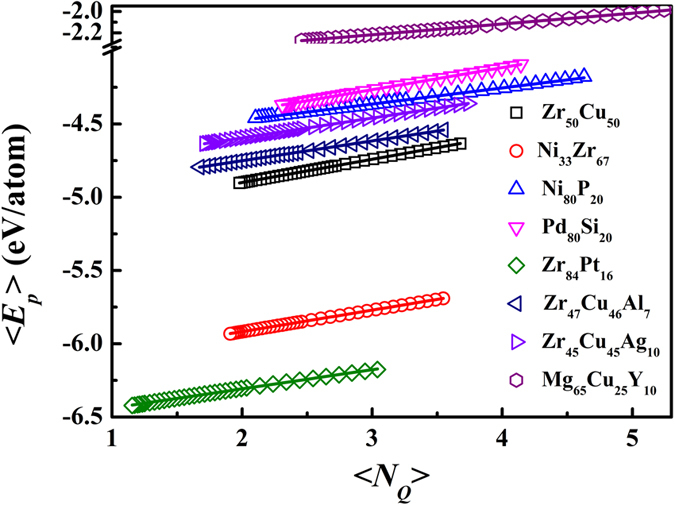
Correlation between <*N*_*Q*_> and per-atom potential energy in eight systems. The solid curves are the linear fittings.

As indicated in Fig. 4, N_*Q*_ displays close correlation with potential energy at the atomic level. In Fig. 5, we investigated the correlation of their average values in eight systems. Strikingly, <*N*_*Q*_> and per-atom potential energy in all the systems shows simply linear correlation, which suggesting that *N*_*Q*_ plays an important role to link atomic structure and thermodynamic properties.

### Correlating QNA with structure relaxation

**Figure 6:**
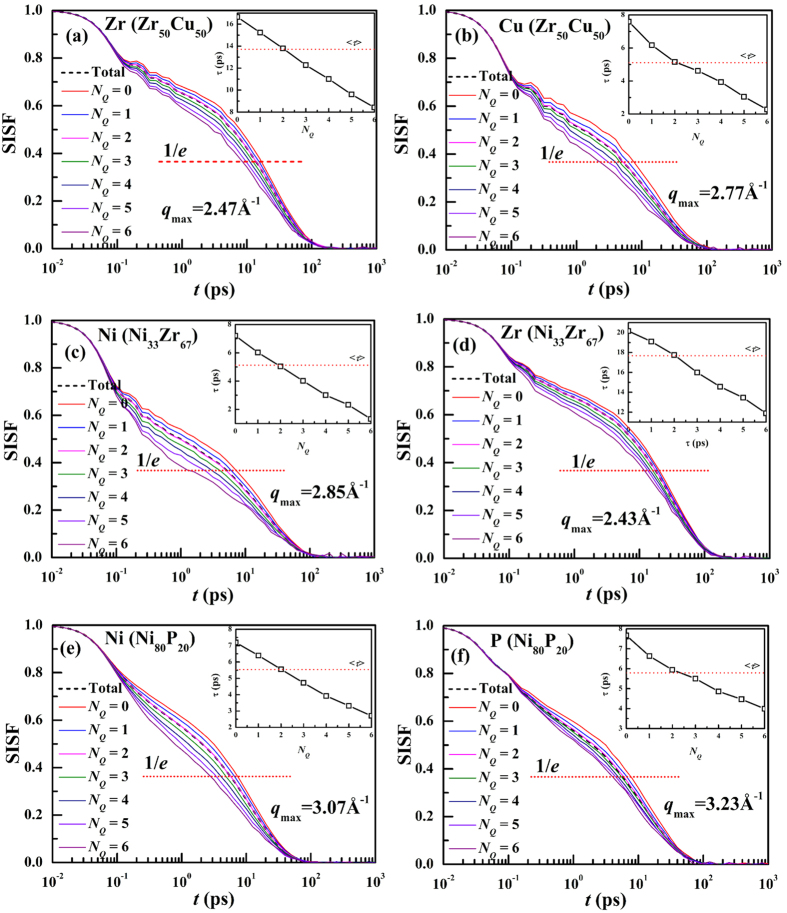
Self-intermediate scattering functions of (**a**) Zr and (**b**) Cu in Zr_50_Cu_50_, (**c**) Ni and (**d**) Zr in Ni_33_Zr_67_ as well as (**e**) Ni and (**f**) P in Ni_80_P_20_ with different *N*_*Q*_ at the initial time at Tg + 150 K. Insets in (**a~f**) are the *N*_*Q*_-dependence of relaxation times and the dash line corresponds to the relaxation time for the element. 100 initial configurations were used to calculate SISFs.

We label all the atoms with different *N*_*Q*_ at initial time. We obtain the structural relaxation time for atoms with the same *N*_*Q*_ by calculating the self-intermediate scattering function (SISF)^23^,


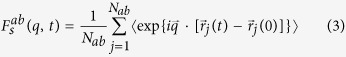


where *N*_*ab*_ is the number of type *a* atoms with *N*_*Q*_ = *b* at *t* = 0, 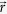 is the position of each atom, 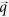 is the wave vector which corresponds to the first peak of the partial structure factor and the average is taken over 100 initial configurations. Figure 6(a,b) display the SISFs of Zr and Cu atoms in Zr_50_Cu_50_ with different *N*_*Q*_. In the long-time relaxation (often called α-relaxation) regime, the SISF with small *N*_*Q*_ decays more slowly compared to that with larger *N*_*Q*_, indicating that atoms with smaller *N*_*Q*_ tend to move slower than those with larger *N*_*Q*_. The α-relaxation time, *τ*_α_, is defined as the time at which the SISF decays to 1/*e* of its initial value. As shown in the insets, for either component of each system, the relaxation time for atoms decreases with increasing *N*_*Q*_. Figure 6(c–f) display the SISFs of Ni and Zr atoms in Ni_33_Zr_67_ as well as Ni and P in Ni_80_P_20_ with different *N*_*Q*_. The results show similar trend to those in Zr_50_Cu_50._ It should be noted that the correlation between *N*_*Q*_ and α-relaxation time for P in Ni_80_P_20_ is much strong. Since the correlation between *N*_*Q*_ and potential energy for P in Ni_80_P_20_ is rather weak shown in Fig. 4(f), it might be reasonable that the correlation between potential energy and dynamic heterogeneity is much weak, at least weaker than *N*_*Q*_.

**Figure 7:**
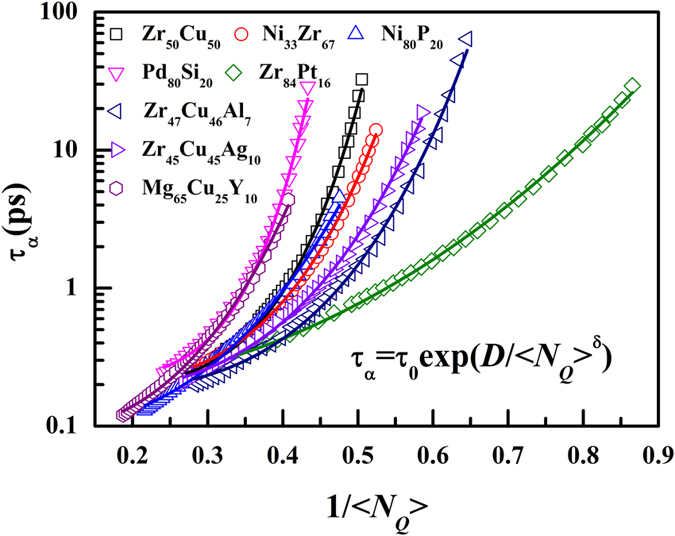
Correlation between <*N*_*Q*_> and α-relaxation time in eight systems. The solid curves are the fittings with equation (4).

Hu *et al.* proposed an equation to link α-relaxation time and <1 − *d*5> in metallic liquids^24^. We found that the equation can also be used to describe the correlation between <*N*_*Q*_> and α-relaxation time:


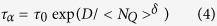


where *τ*_0_ is the relaxation time at infinite liquidus temperature, and δ and *D* are fitting parameters. Figure 7 illustrates the α-relaxation time *τ*_α_ as a function of <*N*_*Q*_> and the fittings of equation (4) for various metallic liquids (*R*^2^ > 0.99 for all the fittings). Remarkably, equation (4) can well describe the relationship between α-relaxation time and <*N*_*Q*_> in these metallic liquids. In addition, δ is fitted to be about 3.36, 3.99, 2.76, 5.26, 2.22, 3.85, 3.49, and 2.90 for Zr_50_Cu_50_, Ni_33_Zr_67_, Ni_80_P_20_, Pd_80_Si_20_, Zr_84_Pt_16_, Zr_47_Cu_46_Al_7_, Zr_45_Cu_45_Ag_10_ and Mg_65_Cu_25_Y_10_ metallic liquids, respectively. Clearly, δ is similar for different systems. δ reflects the sensitivity of α-relaxation time to the change of <*N*_*Q*_>. Therefore, the effect of the <*N*_*Q*_> change on the structure relaxation is similar in different metallic liquids.

## Conclusion

In this work, we study the salient characteristics of structural disorder in metallic liquids employing a new descriptor of local structure, quasi-nearest atom (QNA). By calculating the number of QNAs (*N*_*Q*_) for each atom, we can quantify the degree of atomic packing of an individual atom. From the present analysis, the scaled distribution of *N*_*Q*_, appears to be universal in metallic liquids. The QNA can be correlated with local potential energy and successively with dynamical properties (structural relaxation) at the atomic level. Some straightforward relationships have been proposed to reflect the correlations between QNA and other properties from the macroscopical view. These correlations indicate that the QNA is an important structural identifier that can accurately quantify the local packing and shed light on the structure-property relationship.

Although QNA shows close correlation with some properties in metallic liquids, we noted that it is only a crude measure of how regular the short-range packing order is, and it reflects some information already carried by the Voronoi index. In terms of insight, our results are expected: the larger *N*_*Q*_ is, the worse the local packing order is, the further away from the preferred and best-ordered Kasper polyhedra, the more likely that they are of higher potential energy, and the higher likelihood for them to participate in relaxation and diffusion.

## Method

Classical molecular dynamics simulations are carried out on eight model systems (Zr_50_Cu_50_, Ni_33_Zr_67_, Ni_80_P_20_, Pd_80_Si_20_, Zr_84_Pt_16_, Zr_47_Cu_46_Al_7_, Zr_45_Cu_45_Ag_10_ and Mg_65_Cu_25_Y_10_) of metallic glass-forming liquids through LAMMPS^25^ with the embedded atom method (EAM) potential^26^,^27^,^28^,^29^,^30^,^31^,^32^,^33^. For each model, a cubic simulation box containing 10,000 atoms, with periodic boundary conditions applied in all dimensions, was equilibrated through isothermal-isobaric (*NPT*) simulations (*P* = 0) for enough time to make sure that the potential energy keeps dynamically equilibrated. Pressure and temperature oscillations were controlled through a Nose-Hoover barostat and thermostat, respectively. The equations of motion are integrated using the Verlet algorithm with a time step of 1 fs. The configuration at 2000 K is a random one and Each successive *T* is based on the final of the previous *T*. At each temperature, the initial configuration was relaxed for enough time to make sure that the potential energy of the system reaches dynamically equilibrated. At each temperature of interest, the equilibrated configurations were run for 1 ns (high temperatures) or 10 ns (low temperatures). 1000 configurations were collected for structure analysis and 100 initial configurations were used to calculate dynamical properties. Voronoi polyhedron analysis was performed to describe the atomic structure in metallic liquids. The Voronoi polyhedral index is expressed as <*n*_3_, *n*_4_, *n*_5_, *n*_6_>, where *n*_*i*_ denotes the number of *i*-edged faces of the Voronoi polyhedron. Further analysis based on Voronoi polyhedron was performed to calculating the number of QNAs (*N*_*Q*_) for each atom^32^. More details on the definition of QNA can be found in the supplemental material.

## Additional Information

**How to cite this article**: Pan, S.-P. *et al.* Structural disorder in metallic glass-forming liquids. *Sci. Rep.* **6**, 27708; doi: 10.1038/srep27708 (2016).

## Supplementary Material

Supplementary Information

## Figures and Tables

**Table 1:** The characterized temperature for eight systems.

*Terms*	Zr_50_Cu_50_	Ni_33_Zr_67_	Ni_80_P_20_	Pd_80_Si_20_	Zr_84_Pt_16_	Zr_47_Cu_46_Al_7_	Zr_45_Cu_45_Ag_10_	Mg_65_Cu_25_Y_10_
*T*_*g*_/K	750	700	550	750	900	750	700	550
*T*_VFT_/K	647	556	439	672	761	674	593	448
T^*^/K	162	55	310	14	381	329	243	297
*b*	0.63	0.69	0.56	0.73	1.1	0.69	0.69	0.55

*T*_g_ is the glass transition temperature. *T*_VFT_ is the fitting temperature of Vogel-Fulcher-Tammann equation for relaxation time. T^*^ and *b* are the fitting parameters in equation (1).
