# Supplementary material for: Structural disorder in metallic glass-forming liquids
Source: Sci Rep. 2016 Jun 9;6:27708. doi: 10.1038/srep27708 (PMC4899719; doi:10.1038/srep27708)
Supplement: Supplementary Information [file srep27708-s1.doc]

## Structural disorder in metallic glass-forming liquids

Shao-Peng Pan*^1,2^*^,*^, Shi-Dong Feng*^3^*, Li-Min Wang*^3^,*Jun-Wei Qiao*^1^*,*^2^*, Xiao-Feng Niu*^1^*,*^2^*, Bang-Shao Dong*^4^*, Wei-Min Wang*^5^* and Jing-Yu Qin*^5^*

*^1^**College of Materials Science and Engineering, Taiyuan University of Technology, Taiyuan,* *030024, China*

*^2^Shanxi key laboratory of advanced magnesium-based materials, Taiyuan University of Technology, Taiyuan, 030024, China*

*^3^State Key Laboratory of Metastable Materials Science and Technology, Yanshan University, Qinhuangdao 066004, China*

*^d^Advanced Technology & Materials Co., Ltd., China Iron & Steel Research Institute Group, Beijing100081,China*

*^5^Key Laboratory for Liquid-Solid Structural Evolution and Processing of Materials (Ministry of Education), Shandong University, Jinan 250061, China*

* Correspondence and requests for materials should be addressed to S.P.P. (email: [shaopengpan@gmail.com](mailto:shaopengpan@gmail.com)).


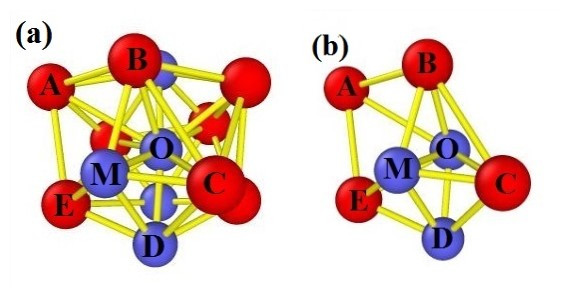


**Fig. S1** The schematic of QNA. (a) A Cu-centered <0,0,12,0> CP in a simulated Cu_50_Zr_50_ metallic liquid. (b) A pentagonal bipyramid extracted from this CP. The blue and red atoms represents Cu and Zr, respectively. The yellow stick between two atoms denotes the nearest correlation of the two atoms. Atoms *A* and *M* belong to a pair of “quasi-nearest” atoms since they are an adjacent pair of the nearest neighbors of atom *O* but not nearest neighbors of each other.

In this supplemental material, we give a detailed description of “quasi-nearest” atom (QNA). In a given configuration, all the nearest neighbors around each atom are determined by Voronoi tessellation method. The Voronoi polyhedral index is expressed as <*n*_3_, *n*_4_, *n*_5_, *n*_6_>, where *n_i_* denotes the number of *i*-edged faces of the Voronoi polyhedron. According to this method, each nearest neighbor of the center atom corresponds to one face of the Voronoi polyhedron. If two Voronoi faces share an edge, the two corresponding atoms are defined as an adjacent pair of atoms. If an adjacent pair of atoms are not the nearest neighbors of each other, we identify these two atoms as a pair of QNAs. Fig. S1(a) depicts a Cu-centered <0,0,12,0> coordination polyhedron (CP) in a simulated Cu_50_Zr_50_ metallic liquids while Fig. S1(b) shows a pentagonal bipyramid extracted from the parent <0,0,12,0> CP of Fig. S1(a). Atoms *A* and *M* are a pair of QNAs because they satisfy the following conditions: (i) both of them are the nearest neighbors of atom *O*; (ii) their corresponding Voronoi faces of the Voronoi polyhedron of Atom *O* share an edge (not shown), thus they are an adjacent pair of atoms; (iii) they are not the nearest neighbors of each other. The coordination polyhedron of Atom *O* can be regarded as its "cage", and the bonding of an adjacent pair of atoms can be regarded as the "stick" of the "cage". If a pair of adjacent atoms are the nearest neighbors of each other, the bonding between them can be regarded to be strong. If a pair of adjacent atoms are not the nearest neighbors of each other (quasi-nearest neighbors), the bonding between them can be regarded to be weak. Thus the pair of QNAs is the weak position of the "cage", where Atom *O* can easily escape from the "cage".
